# Tall women with breast cancer have poorer survival than short women

**DOI:** 10.1101/2024.07.08.24310089

**Published:** 2024-07-09

**Authors:** Steven Lehrer, Peter H. Rheinstein

**Affiliations:** Department of Radiation Oncology, Icahn School of Medicine at Mount Sinai, New York; Severn Health Solutions, Severna Park, Maryland

**Keywords:** breast cancer, height, genetics, risk, survival

## Abstract

**Background:**

Tall women are more likely to develop breast cancer (BC). High Mobility Group AT-Hook 1(HMGA1), an oncofetal protein, plays a role in the progression of breast cancer. Non-coding sequences proximal to HMGA1 contain variants associated with 4.83 cm taller height. In the current study, we used UK Biobank data to examine the relationship of HMGA1 to height, risk, and prognosis of women with breast cancer.

**Methods:**

Our analysis included all subjects with invasive BC that occurred either before or after participant enrollment and were recorded in the UK Biobank database using self-reported data and the International Classification of Diseases (ICD10, ICD9). We divided the subjects into three previously described three height groups: Short (< 155 cm), Medium (155 cm to 175 cm), Tall (> 175 cm). We analyzed the HMGA1 SNP rs41269028, a single nucleotide intron variant, C > T, minor allele frequency 0.044. SNP rs41269028 was previously evaluated in subjects with diabetes.

**Results:**

Height of 9583 women with BC homozygous for the HMGA1 SNP rs41269028 major allele was 162.29 cm ± 6.18. Height of 944 women with BC who were carriers or homozygotes (CT + TT) of the minor allele T was 162.88 cm ± 6.001. This difference was significant (p = 0.005). The effect of height group on survival was significant (p = 0.032, log rank test). Tall women had the poorest survival. The effect of HMGA1 SNP rs41269028 genotype on BC risk (p = 0.602) and survival (p = 0.439, log rank test) was insignificant.

**Conclusion:**

We conclude that HMGA1 influences height, but we were unable to demonstrate that HMGA1 is related to increased incidence or poor prognosis of tall women with breast cancer. We did find that tall women with breast cancer have poorer survival than short women. Our finding that tall women have a worse prognosis is important because it could help the oncologist decide, along with other prognostic factors, whether adjuvant therapy is warranted.

Tall women are more likely to develop breast cancer (BC) [1]. Women who are 176 cm or taller have a 20%–30% higher risk of breast cancer than women who are roughly 155 cm or lower, according to a pooled analysis that included data from 20 prospective cohort studies [2]. The growth spurts tall women experienced as children have been postulated to elevate risk of breast cancer associated with height. Increased hormone levels like IGF-1 or other growth factors can trigger growth spurts. Higher hormone levels and rapid cell proliferation during a growth spurt are thought to influence risk of breast cancer in later life.

High Mobility Group AT-Hook 1(HMGA1), an oncofetal protein, plays a role in the progression of breast cancer [3]. HMGA1 establishes an autocrine loop in invasive triple-negative breast cancer (TNBC) cells, which mediates the migration, invasion, and metastasis of TNBC cells and predicts the onset of metastasis in these patients.

Hawkes et al performed a whole genome sequencing association analysis for height using 333,100 individuals from three datasets: UK Biobank, TOPMed and All of Us. They identified non-coding sequences proximal to HMGA1 containing variants associated with a 4.83 cm taller height [4]. In the current study, we used UK Biobank data to examine the relationship of HMGA1 to height, risk, and prognosis of women with breast cancer.

## Methods

The UK Biobank is a large prospective observational study of men and women with no link to MedWatch. Participants were recruited from across 22 centers located throughout England, Wales, and Scotland between 2006 and 2010 and continue to be longitudinally followed for capture of subsequent health events [5]. This methodology is like that of the ongoing Framingham Heart Study [6], with the exception that the UKB program collects postmortem samples, which Framingham did not.

UK Biobank: has approval from the Northwest Multi-center Research Ethics Committee (MREC) to obtain and disseminate data and samples from the participants, and these ethical regulations cover the work in this study. Written informed consent was obtained from all participants. Details can be found at www.ukbiobank.ac.uk/ethics.

Our UK Biobank application was approved as UKB project 57245 (S.L., P.H.R.). Our analysis included all subjects with invasive BC that occurred either before or after participant enrollment and was recorded in the UK Biobank database using self-reported data and the International Classification of Diseases (ICD10, ICD9).

We divided the subjects into three previously described height groups [1]: Short (< 155 cm), Medium (155 cm to 175 cm), Tall (> 175 cm).

We analyzed the HMGA1 SNP rs41269028, a single nucleotide intron variant, C > T, minor allele frequency 0.044. SNP rs41269028 was previously evaluated in subjects with diabetes [7, 8].

Data processing was performed on Minerva, a Linux mainframe with Centos 7.6, at the Icahn School of Medicine at Mount Sinai. We used PLINK, a whole-genome association analysis toolset, to analyze the UKB chromosome files [9]. Statistical analysis was done with SPSS 26.

## Results

Data from 273,378 women, of which 10,527 were invasive breast cancer cases, was analyzed. Breast cancer patients were aged 60 ± 7 (mean ± SD). 95% of subjects were white British.

Table 1 shows HMGA1 SNP rs41269028 genotype versus height group in 8,327 post-menopausal breast cancer cases. A greater proportion of tall women with breast cancer (11.9%) than short women (6.8%) were carriers or homozygotes (CT + TT) of the minor allele T (p = 0.02, two tail Fisher exact test). No significant effect was present in pre-menopausal women (p = 0.441).

Height of 9583 women with BC homozygous for the HMGA1 SNP rs41269028 major allele (CC) was 162.29 cm ± 6.18. Height of 944 women with BC who were carriers or homozygotes (CT + TT) of the minor allele T was 162.88 cm ± 6.001. This difference was significant (p = 0.005).

**Figure 1.**
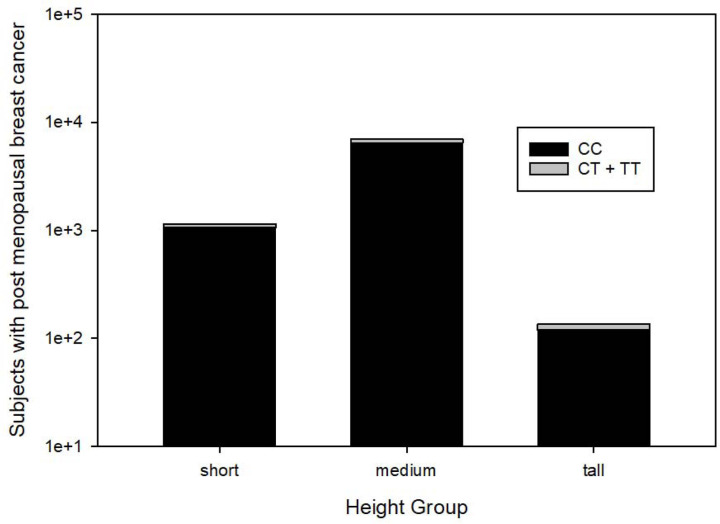
8,327 post-menopausal breast cancer cases stratified by height group and HMGA1 SNP rs41269028 genotype (CC versus CT or TT). Note that tall women have the greatest proportion of carriers or homozygotes (CT + TT) for the minor allele T (p = 0.02, two tail Fisher exact test).

Figure 1 illustrates 8,327 post-menopausal breast cancer patients stratified by height group and HMGA1 SNP rs41269028 genotype (CC versus CT or TT).

Aging is associated with height loss [10]. To correct for this effect, multivariate linear regression was performed on breast cancer cases, height group dependent variable, HMGA1 SNP rs41269028 genotype and age independent variables. The effect of genotype on height groups was significant (B = 0.040, p = 0.002) and independent of the effect of age (B = − 0.005, p < 0.001). In other words, carriers or homozygotes of the minor allele T were taller than homozygotes for the major allele (CC); while older women were shorter than younger women.

**Figure 2.**
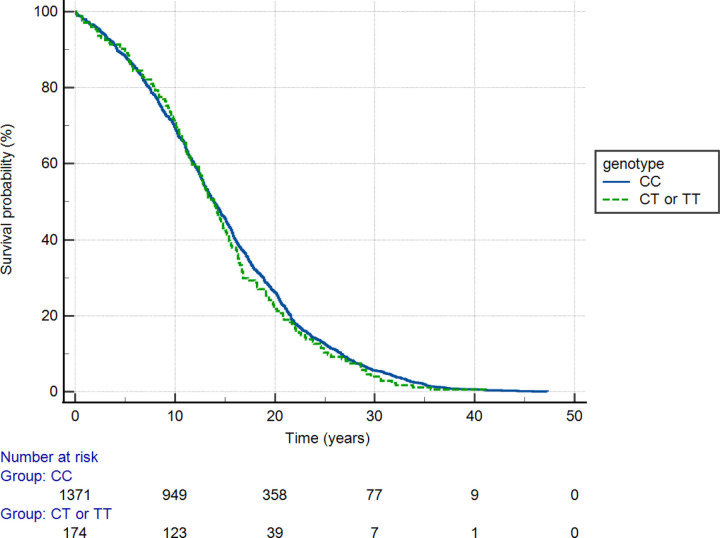
Survival of breast cancer subjects stratified by HMGA1 SNP rs41269028 genotype. The effect of genotype was insignificant (p = 0.439, log rank test).

Figure 2 illustrates survival of breast cancer subjects stratified by HMGA1 SNP rs41269028 genotype. The effect of genotype was insignificant (p = 0.439, log rank test).

**Figure 3.**
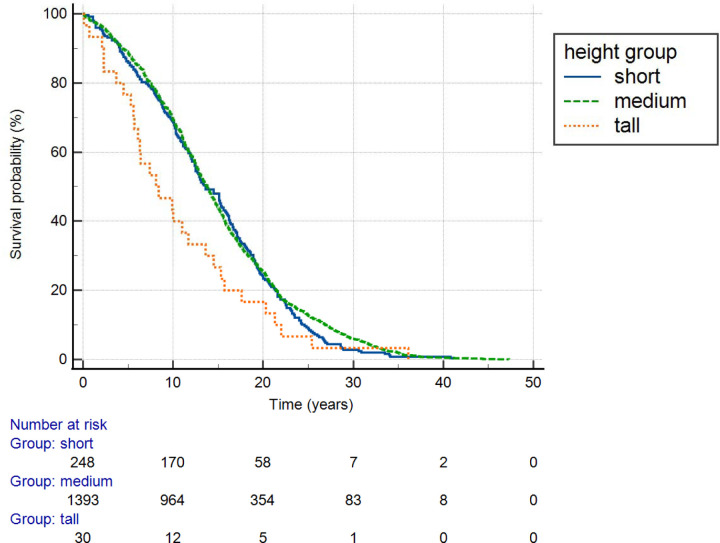
Survival of breast cancer subjects stratified by height group. The effect of height group was significant (p = 0.032, log rank test). Tall women had the poorest survival.

Figure 3 shows survival of breast cancer subjects stratified by height group. The effect of height group was significant (p = 0.032, log rank test). Tall women had the poorest survival.

Table 2 has results of logistic regression, O.R. odds ratio, 228,611 women, breast cancer yes or no dependent variable, age, menopause status, height group, independent variables. Risk of breast cancer was increased in post-menopausal women (O.R. 4.315, p < 0.001). Risk increased with each year of age (O.R. 1.034, p < 0.001). Short women were at decreased risk compared to tall women (O.R. 0.819, p = 0.026). Women of medium height were at decreased risk that was not significant compared to tall women (O.R. 0.929, p = 0.391). HMGA1 SNP rs41269028 had no significant relationship to BC risk (p = 0.602).

## Discussion

Most of the genetic variation linked to complex phenotypes like height is found in non-coding sections of the genome. 99% of the human genome is non-coding, meaning that the great majority of inherited genetic variation is both uncommon and found there. Identifying the uncommon non-coding variation linked to common features and disorders may help uncover new regulatory gene pathways and significantly advance our knowledge of human biology and disease [4].

HMGA1 SNP rs41269028 is an intron variant in a non-coding region of the genome, chromosome 6. Rare variants such as those of HMGA1 are said to confer most of the heredity for height, about 79% [11]. In other words, in a large group of people 79% of height differences are genetic [12].

HMGA1 is a protein that has been found to play a role in the progression of breast cancer. One study suggests that HMGA1 establishes an autocrine loop in invasive triple-negative breast cancer (TNBC) cells, which mediates the migration, invasion, and metastasis of TNBC cells and predicts the onset of metastasis in these patients [13].

HMGA1 promotes breast cancer angiogenesis by supporting the stability, nuclear localization, and transcriptional activity of FOXM1 [14]. FOXM1 is an oncogenic transcription factor that is greatly upregulated in breast cancer and many other cancers where it promotes tumorigenesis, cancer growth and progression. It is expressed in all subtypes of breast cancer and is the factor most associated with risk of poor patient survival, especially in (TNBC).

Our study has weaknesses. We did not have tumor size, histology, grade, or hormone receptor status. We did not have the recently released UKBB whole genome sequence data for HMGA1 that Hawkes et al used [4, 15]. Instead, we evaluated imputed genotypes from UKB data field 22828 [16]. We found that HMGA1 SNP rs41269028 minor allele T carriers (CT) and homozygotes (TT) were significantly taller, but the effect size was small, 0.59 cm, not the 4.83 cm that Hawkes et al reported [4].

## Conclusion

We conclude that HMGA1 influences height, but we were unable to demonstrate that HMGA1 is related to increased incidence or poor prognosis of tall women with breast cancer. Our finding that tall women have a worse prognosis is important because it could help the oncologist decide, along with other prognostic factors, whether adjuvant therapy is warranted.

## Figures and Tables

**Table 1.** HMGA1 SNP rs41269028 genotype versus height group in 8,327 post-menopausal breast cancer cases. A greater proportion of tall women with breast cancer (11.9%) than short women (6.8%) were carriers or homozygotes (CT + TT) of the minor allele T (p = 0.02, two tail Fisher exact test).

		CC	CT or TT	
short	Count	1068	78	1146
	% within height group	93.2%	6.8%	100%
medium	Count	6414	632	7046
	% within height group	91.0%	9.0%	100%
tall	Count	119	16	135
	% within height group	88.1%	11.9%	100%
total	Count	7601	726	8327
	% within height group	91.3%	8.7%	100%

**Table 2.** Logistic regression, L.B. lower bound, U.B. upper bound, O.R. odds ratio, 228,611 women, breast cancer yes or no dependent variable, age, menopause status, height group, independent variables. Risk of breast cancer was increased in post-menopausal women (O.R. 4.315, p < 0.001). Risk increased with each year of age (O.R. 1.034, p < 0.001). Short women were at decreased risk compared to tall women (O.R. 0.819, p = 0.026). Women of medium height were at decreased risk that was not significant compared to tall women (O.R. 0.929, p = 0.391). HMGA1 SNP rs41269028 had no significant relationship to BC risk (p = 0.602).

	95% L.B.	O.R.	95% U.B.	p value
menopause	3.866	4.315	4.815	<0.001
age	1.030	1.034	1.038	<0.001
short	0.689	0.819	0.973	0.026
medium	0.788	0.929	1.095	0.391
HMGA1	0.908	0.980	1.058	0.602
